# *CEBPD* may function as a molecular indicator of fibrotic severity and negative regulator of fibrosis in uterine leiomyoma through regulating EMT progression

**DOI:** 10.3389/fphar.2026.1839523

**Published:** 2026-05-19

**Authors:** Ran Duan, Xiaole Zuo, Qing Zhang, Jinhao Wang, Dongmei Xu, Min Li, Li Qu, Botao Zhao

**Affiliations:** 1 Department of Clinical Laboratory, Taian Maternal and Child Healthcare Hospital, Taian, Shandong, China; 2 Department of Blood Transfusion, Taian Maternal and Child Healthcare Hospital, Taian, Shandong, China; 3 Department of Obstetrics, Taian Maternal and Child Healthcare Hospital, Taian, Shandong, China; 4 Department of Gynecology, Taian Maternal and Child Healthcare Hospital, Taian, Shandong, China

**Keywords:** *CEBPD*, diagnostic biomarker, EMT progression, fibrosis, single-cell RNA sequencing, uterine leiomyoma

## Abstract

**Objective:**

To investigate the expression pattern, diagnostic value, and molecular mechanism of CCAAT/enhancer-binding protein delta (*CEBPD*) in uterine leiomyoma (ULM)-associated fibrosis, and to identify novel diagnostic biomarkers and therapeutic targets for ULM.

**Methods:**

We analyzed transcriptional profiles of two independent ULM cohorts (GSE64763, GSE95101) to screen differentially expressed genes and enriched pathways. The diagnostic performance of *CEBPD* was evaluated via receiver operating characteristic (ROC) curve analysis. Single-cell RNA sequencing (scRNA-seq) data were used to map *CEBPD*’s cellular distribution in the ULM microenvironment, with pseudotime and correlation analyses to explore its association with epithelial-mesenchymal transition (EMT) and fibrosis. Histological validation was performed using H&E and double immunofluorescence staining in clinical ULM specimens.

**Results:**

*CEBPD* was significantly downregulated in ULM tissues, with enrichment in immune-related signaling pathways. It exhibited robust diagnostic accuracy for ULM, with area under the curve (AUC) values of 0.878 (95% CI: 0.783–0.973) and 0.858 (95% CI: 0.735–0.981) in the two cohorts. scRNA-seq identified 8 cell subsets in the ULM microenvironment, and *CEBPD* expression was dynamically and negatively correlated with key fibrotic mediators (*COL1A1*, *COL1A2*, *COL3A1*, all *p* < 0.001) and EMT-related factors (*ZFP36*, *KLF2*, *NR4A1*, all *p* < 0.001). Co-localization of CEBPD and T lymphocyte marker CD3E was confirmed in clinical specimens.

**Conclusion:**

*CEBPD* emerges as a potential prognostic biomarker for uterine fibroids. Mechanistically, its dynamic expression is strongly associated with the fibrotic cascade and inversely correlated with EMT progression as well as the expression of major extracellular matrix genes. These findings suggest that *CEBPD* may be a key regulatory factor in fibroid pathogenesis, offering a promising molecular target that warrants further functional validation in clinical management.

## 1 Introduction

Uterine fibroids (leiomyomas) stand as the most frequently diagnosed benign neoplasms within the female reproductive tract. Pathologically, these tumors are defined by the aberrant expansion of smooth muscle bundles coupled with the massive accumulation of extracellular matrix (ECM), making them fundamentally a fibrotic disorder (Serge et al., 2024). The prevalence is strikingly high, impacting approximately 20%–50% of women during their childbearing years (Zhuanzhuan et al., 2026). The clinical burden is substantial, as patients frequently suffer from heavy menstrual bleeding, pelvic discomfort, and impaired fertility, all of which severely diminish their overall wellbeing (Emma E et al., 2023).

At present, clinical management heavily relies on surgical extirpation or pharmacological therapies (Ganesh et al., 2026). However, surgical procedures are inherently invasive, and prolonged medication regimens are frequently accompanied by undesirable adverse events. Furthermore, the absence of highly sensitive, specific biomarkers hampers the feasibility of early detection and the deployment of precision medicine strategies (Ombretta et al., 2023). Consequently, unraveling the precise molecular drivers behind fibroid tumorigenesis and fibrogenesis is imperative for the discovery of innovative diagnostic tools and therapeutic vulnerabilities (Qiwei and Ayman, 2023). Among the multitude of molecular factors driving fibrotic diseases, the transcription factor CCAAT/enhancer-binding protein delta (*CEBPD*) has increasingly drawn scientific interest owing to its regulatory roles in critical cellular events, including apoptotic cascades, cellular differentiation, and inflammatory responses (Mayra et al., 2024). Despite this, the precise mechanistic contribution of *CEBPD* to the fibrotic pathology of uterine fibroids is still poorly defined.

Recent investigations have hinted at a crosstalk between *CEBPD* and principal fibrogenic networks, particularly those governing immune modulation and the epithelial-mesenchymal transition (EMT) (Minxuan et al., 2023). Given that EMT is inextricably linked to the aberrant smooth muscle proliferation and extracellular matrix overproduction seen in these tumors, *CEBPD* emerges not merely as a potential core regulator of fibroid progression but also as a highly viable candidate for targeted pharmacological therapies. To address these knowledge gaps, the current investigation utilizes an integrative multi-omics strategy, synthesizing findings from bulk transcriptomics, single-cell RNA sequencing (scRNA-seq), and empirical histological assays (such as H&E and immunofluorescence co-staining). This comprehensive framework is highly effective for extracting crucial regulatory elements from publicly accessible leiomyoma cohorts, facilitating an in-depth, high-resolution dissection of their functions within the heterogeneous tumor microenvironment. Moreover, validating the spatial relationship between specific molecular targets—namely *CEBPD* and *CD3E*—provides a deeper, more contextualized perspective on the fibrotic mechanisms at play.

Specifically, we sought to map the primary genetic signatures driving fibroid fibrosis, evaluate the clinical diagnostic value of *CEBPD*, profile its expression across various uterine cell types, and clarify its functional ties to both EMT dynamics and fibrogenesis at the subpopulation level. Ultimately, deploying this combined computational and experimental workflow bridges critical bioinformatics predictions with tangible biological evidence. Uncovering the functional landscape of *CEBPD* provides critical insights into the molecular etiology of fibroid-associated fibrosis, paving the way for advanced diagnostic modalities and innovative therapeutic interventions aimed at improving clinical outcomes for this widespread gynecological condition.

## 2 Materials and methods

### 2.1 Data acquisition

Transcriptomic arrays (specifically GSE64763, GSE95101, and GSE162122), encompassing paired uterine fibroid and normal myometrial specimens, were retrieved from the Gene Expression Omnibus (GEO) repository (Table 1).

**TABLE 1:** Supplementary baseline summary table.

Dataset ID	Detection type	Platform	Total included samples	Grouping (case/control)	Patient inclusion characteristics
GSE64763	Bulk transcriptome microarray	Affymetrix human genome U133A 2.0 array	79	23 ULM/29 NL	Pre- to peri-menopausal females, no preoperative hormone therapy
GSE95101	Bulk transcriptome microarray	Illumina human WG-6 v3.0 BeadChip	38	20 ULM/18 NL	Pre-menopausal symptomatic ULM patients, no preoperative hormone therapy
GSE162122	scRNA-seq	Illumina NovaSeq 6000 (10X genomics)	10	5 ULM/5 NL	Pre-menopausal females, MED12-mutant ULM, no preoperative hormone therapy

ULM = uterine leiomyoma; NL = normal myometrium.

For higher-resolution cellular profiling, scRNA-seq datasets derived from equivalent human tissues were also collected to execute gene expression, cell clustering, and functional correlation assessments.

### 2.2 Analysis of differentially regulated genes and pathway enrichment

To pinpoint differentially expressed genes (DEGs) distinguishing fibroids from healthy controls in the GSE64763 and GSE95101 cohorts, we deployed the limma package within the R environment (version 4.2.1). Statistical significance was strictly defined by an adjusted *p*-value (FDR) below 0.05 alongside a log2 fold-change threshold exceeding 1. Subsequent Gene Set Enrichment Analysis (GSEA) was executed utilizing clusterProfiler to map these DEGs to relevant biological pathways, sorted based on the corrected P-value, and retained the top 15 pathways.

### 2.3 Evaluation of diagnostic efficacy of *CEBPD*

The capability of *CEBPD* to effectively discriminate leiomyoma tissues from normal counterparts was quantified via Receiver Operating Characteristic (ROC) analytics. Utilizing the pROC R package, we constructed ROC curves based on the expression metrics from the GSE64763 and GSE95101 series, computing the Area Under the Curve (AUC) along with its 95% confidence intervals (CI) to determine diagnostic precision.

### 2.4 Immune cell infiltration and correlation analysis

The CIBERSORT computational framework was utilized to deconvolute and estimate the relative abundances of infiltrating immune cells within the targeted GEO datasets. Differences in the fraction of M2-polarized macrophages and resting mast cells between the pathological and normal groups were statistically evaluated using the Wilcoxon rank-sum test. Furthermore, Pearson correlation metrics were applied to uncover any significant associations (*p* < 0.05) between *CEBPD* transcript levels and these specific immune cell populations.

### 2.5 Protein-Protein Interaction (PPI) network and subcellular localization

A Protein-Protein Interaction (PPI) network centered around *CEBPD* was generated relying on the STRING database and subsequently rendered using Cytoscape. To determine *CEBPD*’s spatial distribution, predictions from the Human Protein Atlas (HPA) were gathered and empirically corroborated through immunofluorescence assays, which established its primary residence within the nucleoplasm.

### 2.6 scRNA-seq data processing and cell clustering

scRNA-seq downstream analyses were primarily executed using the Seurat framework in R. Initial quality assurance steps excluded low-quality cells based on the following criteria: nFeature_RNA spanning 200 to 5,000, and nCount_RNA bounded between 500 and 20,000. A mitochondrial transcript proportion threshold of <20% was applied; this relatively permissive cutoff was specifically selected to preserve biologically viable stromal and smooth muscle cell populations that may exhibit elevated baseline stress signatures following the robust enzymatic and mechanical dissociation required for dense, fibrotic leiomyoma tissues (Supplementary File S1). Following data normalization and identification of highly variable features, we applied the Harmony algorithm (via the harmony R package) to integrate the datasets and effectively mitigate batch effects across different patient samples. The resulting Harmony embeddings were subsequently utilized for Uniform Manifold Approximation and Projection (UMAP) dimensionality reduction, and discrete cell clusters were designated relying on established lineage markers, including *ACTA2* for SMCs, *DCN* for fibroblasts, *CD3D* for T cells, among others (*NKG7*, *PECAM1*, *CD163*, *EPCAM*). Subpopulation frequencies were then quantified and graphically represented.

### 2.7 Analysis of *CEBPD* expression characteristics

By leveraging scRNA-seq repositories from the Human Cell Atlas, we profiled *CEBPD* expression across various endometrial and immune cell compartments. Within the fibroid scRNA-seq landscape, *CEBPD* metrics were transformed into log2 (TPM+1) z-scores, allowing for spatial mapping across clusters via violin and UMAP plots. Expression disparities between fibrotic and normal myometrial subsets were determined through Wilcoxon rank-sum testing.

### 2.8 Correlation analysis of CEBPD with fibrosis and EMT progression

To map the transitional states of *CEBPD* along the fibrotic and EMT axes, pseudotime trajectory inference was modeled using the Monocle 2 algorithm based on DDRTree dimensionality reduction. The root state (pseudotime = 0) was biologically anchored to the cell cluster exhibiting the lowest EMT and fibrosis-related module scores, representing a more quiescent stromal state prior to fibrotic transdifferentiation. Discrete EMT scores, acting as a pseudotime proxy, were calculated using the single-sample Gene Set Enrichment Analysis (ssGSEA) algorithm via the GSVA R package. The established EMT gene set utilized for this scoring was the HALLMARK_EPITHELIAL_MESENCHYMAL_TRANSITION pathway, sourced from the MSigDB Hallmark gene sets (version h. all.v2023.2. Hs.symbols.gmt). The interplay between *CEBPD* levels and discrete EMT scores across various cell clusters was quantified using Pearson correlations. Heatmap visualizations were constructed to display expression gradients of *CEBPD* alongside classical collagen markers (e.g., *COL1A1*, *COL1A2*, *COL3A1*, *ACTA2*) and EMT drivers (e.g., *ZFP36*, *KLF2*, *NR4A1*) over pseudotime. Additionally, the CellChat toolkit facilitated the mapping of intercellular communication networks, specifically targeting collagen signaling to pinpoint central sender, receiver, mediator, and influencer cells (code was listed in Supplementary File S2).

### 2.9 HE staining and immunofluorescence co-staining for CD3E

Clinical leiomyoma and paired adjacent normal myometrium specimens were collected from surgical patients at Tai’an Maternal and Child Health Hospital, with all procedures strictly adhering to the Institutional Ethics Committee protocols and the Declaration of Helsinki (Ethics Approval No. TAFY-LL-KYLX202307). Written informed consent was obtained from all enrolled participants prior to surgery. A total of 6 formalin-fixed paraffin-embedded (FFPE) tissue specimens were collected from 3 patients with pathologically confirmed uterine leiomyoma who underwent hysterectomy. For each patient, one uterine leiomyoma (ULM) tissue specimen and one paired adjacent normal myometrium (ANM) tissue specimen were collected simultaneously. ULM specimens were sampled from the central viable area of the tumor, avoiding necrotic and hemorrhagic regions; paired ANM specimens were collected at a site at least 2 cm away from the leiomyoma pseudocapsule, with no visible tumor nodules under macroscopic observation, and histologically confirmed to have normal smooth muscle structure, no abnormal proliferation, and no microscopic leiomyoma lesions. Inclusion Criteria: Pre-menopausal female patients aged 30–50 years; no hormone therapy, radiotherapy, or chemotherapy received within 3 months before surgery; no history of malignant tumors, endometriosis, adenomyosis, or autoimmune diseases; all specimens were histologically confirmed by two independent board-certified gynecological pathologists. Exclusion Criteria: Patients complicated with gynecological malignancies, acute or chronic pelvic inflammatory disease, severe liver and kidney dysfunction, or preoperative hormone exposure within 3 months were excluded from this study. Tissue processing involved 4% paraformaldehyde fixation, paraffin embedding, and sectioning at a 5 μm thickness. Following routine Hematoxylin and Eosin (H&E) staining for architectural validation, immunofluorescence was conducted. Sections were probed with anti-CEBPD (1:200, Abcam) and anti-CD3E (1:150, Cell Signaling Technology) primary antibodies, detected via Alexa Fluor-conjugated secondaries, and counterstained using DAPI. High-resolution imaging was captured on a laser scanning confocal platform, with colocalization metrics processed via ImageJ.

### 2.10 Statistical analysis

All computational and statistical operations were executed within R (v4.2.1) and GraphPad Prism (version 9.0). Data for continuous variables were summarized as the mean ± standard deviation. Group-wise comparisons utilized the Wilcoxon rank-sum test, whereas linear dependencies were probed via Pearson correlation. Statistical thresholds were established at *p* < 0.05 for significance and *p* < 0.001 for extreme statistical significance.

## 3 Results

### 3.1 Transcriptomic profiling reveals downregulation of *CEBPD* and enrichment of pro-inflammatory cascades

**FIGURE 1:**
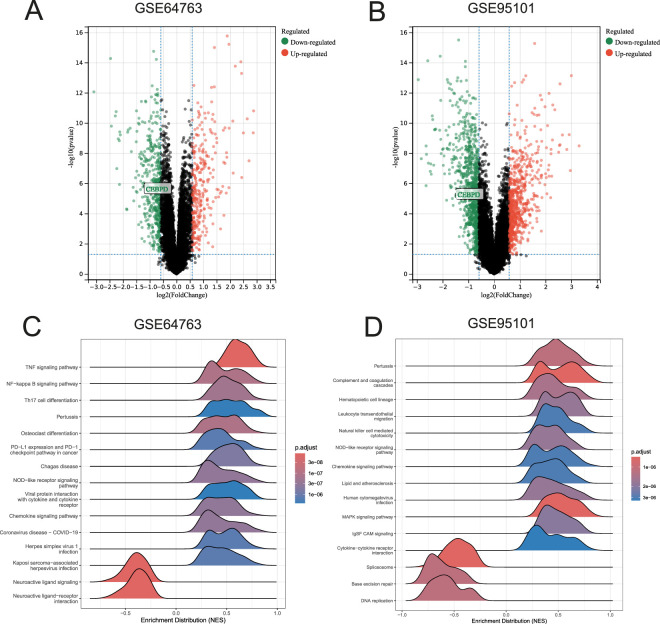
Identification of differentially expressed genes and pathway enrichment in uterine leiomyoma. **(A,C)** Volcano plots illustrating the differentially expressed genes in the GSE64763 and GSE95101 datasets, respectively. The downregulation of *CEBPD* in fibroid tissues is highlighted. **(B,D)** Bar charts representing the top enriched pathways identified via GSEA based on the NES.

Systematic evaluation of two independent transcriptomic arrays (GSE64763 and GSE95101) revealed a profound and consistent suppression of *CEBPD* transcription in leiomyoma tissues compared to healthy myometrial controls (log2FC < −1, −log10(*p*) > 4) (Figures 1A,C). Subsequent GSEA indicated that this downregulation was intrinsically linked to key inflammatory and immune-regulatory networks. Notably, the TNF, NF-κB, and NOD-like receptor signaling pathways were significantly enriched (NES > 0, *p*. adjust < 1e-06) (Figures 1B,D), concurrent with heightened leukocyte transendothelial migration and natural killer cell-mediated cytotoxicity (Supplementary Files S3, S4).

### 3.2 *CEBPD* demonstrates robust diagnostic efficacy in uterine leiomyoma

**FIGURE 2:**
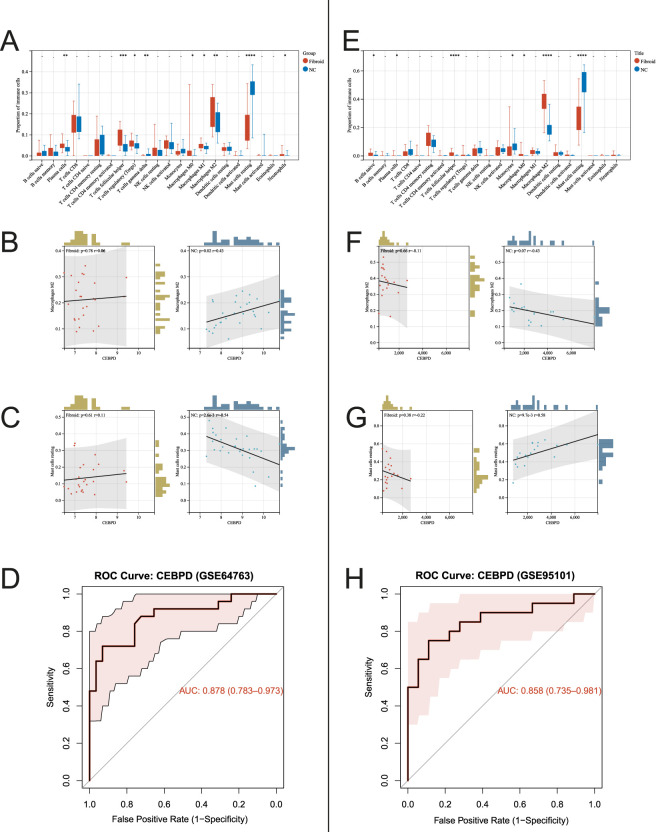
Immune cell infiltration profiles and diagnostic efficacy of CEBPD. **(A,E)** Boxplots showing the differential proportion of various infiltrating immune cell types between fibroid and NC tissues across datasets. **(B,C,F,G)** Scatter plots depicting the Pearson correlation between CEBPD expression and the infiltration levels of M2 macrophages and resting mast cells in both the fibroid and NC groups. **(D,H)** ROC curves evaluating the diagnostic accuracy of CEBPD in distinguishing uterine leiomyoma from normal tissues in the GSE64763 (AUC = 0.878) and GSE95101 (AUC = 0.858) datasets.

Analysis of the tumor immune microenvironment highlighted a significant accumulation of M2-polarized macrophages within fibrotic tissues (*p* < 0.05), whereas resting mast cells were predominantly localized in the normal control (NC) group (*p* < 0.05) (Figures 2A,E). In healthy myometrium, *CEBPD* transcript levels exhibited a positive correlation with M2 macrophages (GSE64763: r = 0.43, *p* = 0.02) and distinct associations with resting mast cells; however, these regulatory correlations were uncoupled in the pathological state (Figures 2B,C,F,G). Importantly, ROC analysis validated the substantial clinical diagnostic potential of *CEBPD*, yielding exceptional AUC values of 0.878 and 0.858 in the GSE64763 and GSE95101 datasets, respectively (Figures 2D,H).

### 3.3 Spatial localization and cellular expression hierarchy of *CEBPD*

**FIGURE 3:**
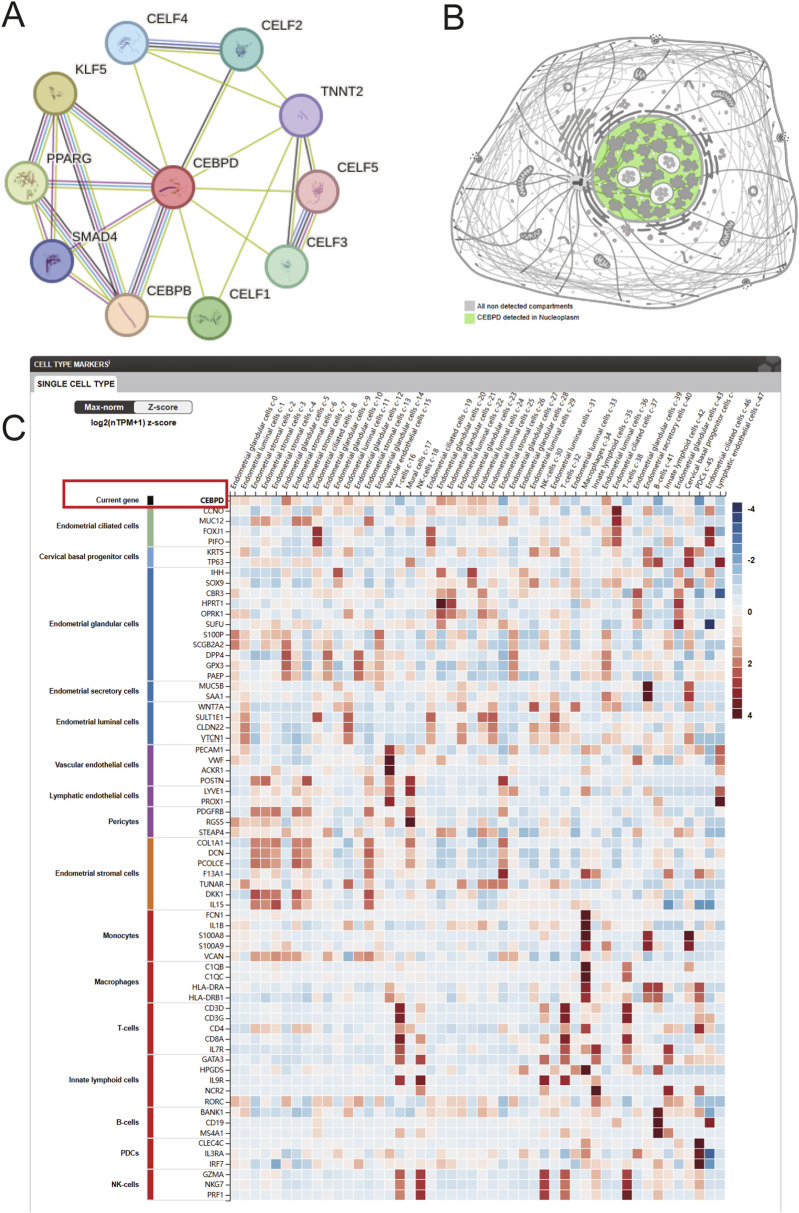
PPI network and expression characteristics of *CEBPD*. **(A)** The PPI network centered on CEBPD, demonstrating its interactions with transcription factors and RNA-binding proteins (e.g., SMAD4, PPARG, KLF5). **(B)** Subcellular localization diagram confirming that CEBPD is predominantly detected in the nucleoplasm. **(C)** Heatmap showing the log2 (nTPM+1) z-score expression profiles of *CEBPD* and canonical cell type markers across various human endometrial single-cell types.

To further understand the molecular role of *CEBPD*, a reconstructed PPI network illustrated its extensive interactions with critical transcription factors (e.g., *CEBPB*, *KLF5*) and RNA-binding proteins, underscoring its pivotal role in transcriptional modulation (Figure 3A). The data in HPA confirmed that *CEBPD* was strictly confined to the nucleoplasm (Figure 3B). Furthermore, scRNA-seq profiling of the human endometrium uncovered a distinct expression hierarchy, with *CEBPD* being predominantly enriched within endometrial epithelial and stromal cellular lineages (Figure 3C).

### 3.4 scRNA-seq clustering unveils the cellular heterogeneity of the fibroid microenvironment

**FIGURE 4:**
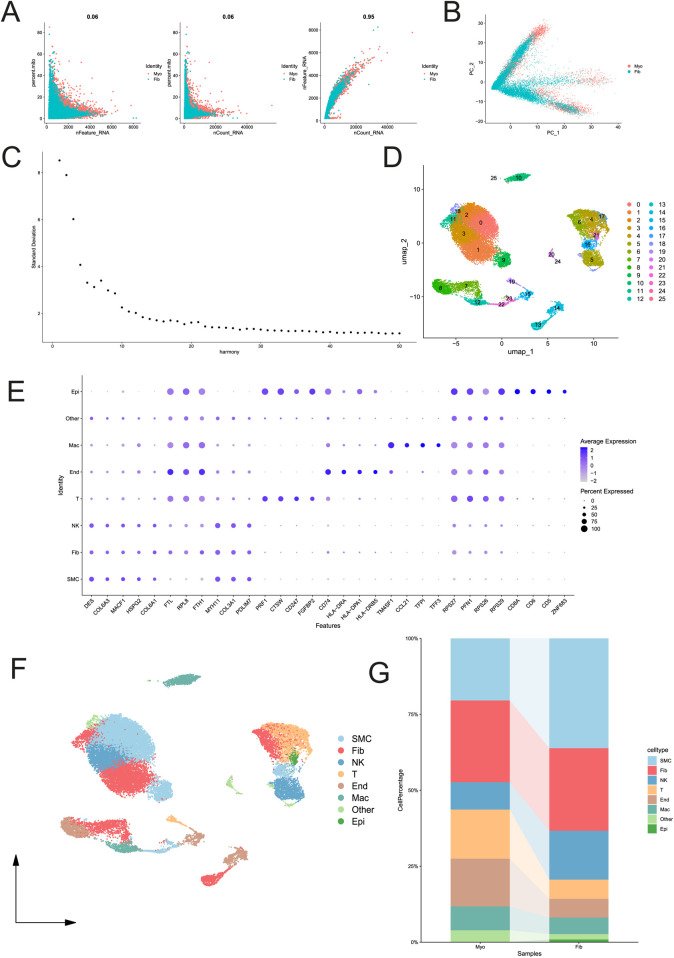
Quality control and single-cell RNA sequencing (scRNA-seq) clustering of the tumor microenvironment. **(A–C)** Quality control metrics for the scRNA-seq data, displaying the correlation between nCount_RNA, nFeature_RNA, and mitochondrial gene percentages. **(D)** Uniform Manifold Approximation and Projection (UMAP) plot revealing 8 distinct cell subpopulations: SMC, Fib, NK, T, End, Mac, Other, and Epi. **(E)** Dot plot illustrating the average expression and expression percentage of selected marker genes across the identified cell clusters. **(F)** UMAP visualization split by sample origin (Myo vs. Fib). **(G)** Stacked bar plot showing the cell percentage distribution of each subpopulation in normal myometrial and fibroid samples.

Transitioning to the single-cell level, rigorous quality filtration and UMAP dimensionality reduction successfully delineated the leiomyoma microenvironment into eight distinct subpopulations: SMCs, fibroblasts (Fib), NK cells, T cells, endothelial cells, macrophages, epithelial cells, and an undefined “other” cluster (Figure 4).

### 3.5 *CEBPD* expression dynamics inversely correlate with EMT and fibrosis trajectories

**FIGURE 5:**
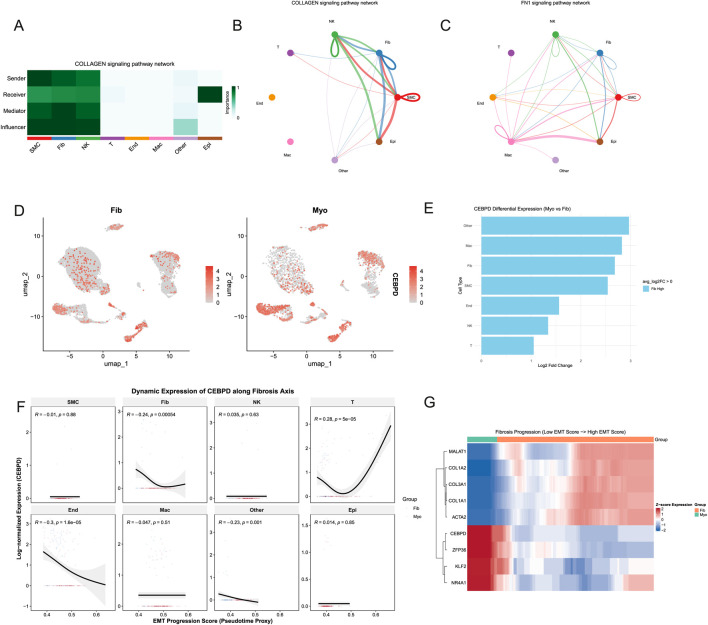
Cell-cell communication and dynamic regulation of *CEBPD* along the fibrosis progression axis. **(A)** Heatmap evaluating the relative importance of different cell types as senders, receivers, mediators, and influencers within the COLLAGEN signaling pathway network. **(B,C)** Circle plots showing the intercellular communication networks for the COLLAGEN pathway among different cell clusters. **(D)** UMAP plots depicting the distinct *CEBPD* expression levels in Fib and Myo groups. **(E)** Bar plot showing the log2 fold change of *CEBPD* differential expression (Myo vs. Fib) across various cell types. **(F)** Line graphs mapping the dynamic expression of *CEBPD* against the EMT progression score (pseudotime proxy) in key cell subpopulations, revealing a significant negative correlation in Fibroblasts (R = −0.24, *p* < 0.001). **(G)** Heatmap visualizing the expression patterns of *CEBPD* alongside EMT-related (*ZFP36*, *KLF2*, *NR4A1*) and collagen-related (*COL1A1*, *COL1A2*, *COL3A1*, *ACTA2*) genes as fibrosis progresses from low to high EMT scores.

Intercellular communication modeling targeting collagen signaling revealed SMCs as the dominant signaling hub, with fibroblasts acting as crucial networking bridges between SMCs and immune cells (Figures 5A–C). UMAP visualization demonstrated that *CEBPD* was significantly upregulated within the fibroblast subset relative to other cell types (Figures 5D,E). Crucially, pseudotime trajectory analysis revealed that as cells advanced along the EMT/fibrosis axis, *CEBPD* expression in fibroblasts exhibited a robust inverse correlation with the EMT progression score (R = −0.24, *p* < 0.001), while a contrasting positive correlation was observed in T cells (R = 0.28, *p* < 0.001) (Figure 5F). Heatmap clustering further corroborated that escalating EMT scores perfectly aligned with enhanced pro-fibrotic collagen expression and the concurrent suppression of *CEBPD*, strongly implicating it as a pharmacological negative regulator of fibrogenesis (Figure 5G).

### 3.6 CEBPD nuclear expression within CD3E-Positive T lymphocytes and its association with local immune responses

**FIGURE 6:**
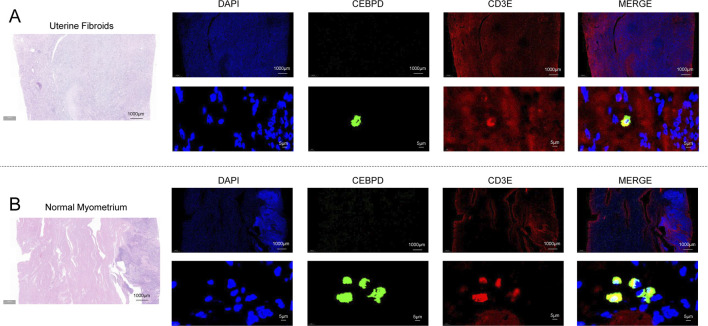
Dual immunofluorescence staining detects CEBPD nuclear expression within CD3E-positive T lymphocytes in human uterine leiomyoma (ULM) and paired normal myometrial tissues. **(A)** Representative dual immunofluorescence staining images of human ULM tissues. Nuclei were counterstained with DAPI (blue), CEBPD was labeled with green fluorescence, and the T lymphocyte-specific marker CD3E was labeled with red fluorescence via corresponding fluorescent secondary antibodies. The merged high-magnification image confirms clear nuclear expression of CEBPD within CD3E-positive infiltrating T lymphocytes in ULM tissues. **(B)** Representative images of dual immunofluorescence staining in paired human normal myometrial tissues, with the same staining protocol and panel layout as described above. Scale bars: 1,000 μm for low-magnification panoramic views, 5 μm for high-magnification detailed views.

Routine H&E staining was performed on the clinical validation cohort specimens, which histopathologically confirmed the typical morphological features of uterine leiomyoma (disordered proliferation of smooth muscle bundles, massive extracellular matrix deposition) and normal myometrium (regularly arranged smooth muscle bundles). Subsequent dual immunofluorescence mapping confirmed that CEBPD nuclear expression was observed within CD3E-positive T lymphocytes, providing empirical evidence that CEBPD may modulate local fibrotic responses through regulation of the T-cell immune compartment (Figure 6).

## 4 Discussion

Uterine leiomyomas, commonly referred to as fibroids, represent the most prevalent benign tumors in the female reproductive system, affecting a significant proportion of women globally, particularly during their reproductive years (Malcolm G et al., 2025).

Their incidence varies across different ethnicities, with higher prevalence rates reported among Black women (Oliwia W et al., 2022).

While many women with uterine leiomyomas remain asymptomatic, approximately one-third experience debilitating symptoms such as pelvic pain, abnormal bleeding, and infertility, which can lead to considerable morbidity and even the necessity for surgical interventions like hysterectomy (Alba et al., 2021; Shikha et al., 2023). Despite the substantial health burden posed by these tumors, current diagnostic and therapeutic options remain limited, emphasizing the need for innovative approaches to understand their underlying molecular mechanisms and to identify potential non-invasive diagnostic biomarkers and targeted therapies (Pooja, 2025).

In this study, we focused on the transcription factor *CEBPD* (CCAAT/Enhancer Binding Protein Delta) due to its implicated roles in inflammatory processes, cell differentiation, and apoptosis, which may influence the pathophysiology of uterine leiomyomas (Sharad et al., 2025). Utilizing a comprehensive multi-omics approach, we aimed to elucidate the expression patterns of *CEBPD* at single-cell resolution, assess its diagnostic potential, and explore its functional correlations with immune cell infiltration and fibrosis progression within the tumor microenvironment. Key findings from our analysis revealed that *CEBPD* is consistently downregulated in leiomyoma tissues, exhibiting high diagnostic accuracy, and its expression is intricately linked to immune-related pathways and specific immune cell populations (Subhash C et al., 2026). These insights lay the groundwork for further discussion on the implications of *CEBPD* in both the diagnostic and therapeutic realms for uterine leiomyomas.

We investigated the role of *CEBPD* in uterine leiomyoma, revealing its consistent downregulation across multiple datasets. This downregulation was significantly associated with pathways related to inflammation and immune responses, such as TNF signaling and NF-κB pathways, indicating that *CEBPD* may play a pivotal role in the disease’s pathogenesis (Spek et al., 2021). The consistent findings across independent datasets strengthen the argument for *CEBPD* as a core dysregulated transcription factor in leiomyoma, while also raising questions regarding the biological mechanisms that underlie these expression patterns. For instance, the biological plausibility of how *CEBPD* regulates immune-related pathways warrants further exploration, particularly in relation to the fibrotic microenvironment observed in leiomyoma. Notably, the high diagnostic accuracy of *CEBPD* expression for distinguishing uterine leiomyoma from normal tissue positions it as a potential biomarker for clinical applications. The ROC analysis demonstrated robust AUC values, suggesting that *CEBPD* could substantially aid in patient stratification and diagnosis (Yan et al., 2024).

Future studies should evaluate the practicality of measuring *CEBPD* expression in clinical samples, considering both tissue-based and blood-based approaches. Additionally, combining *CEBPD* with other biomarkers may enhance diagnostic precision, offering a more comprehensive diagnostic tool for uterine leiomyoma. The analysis of immune cell infiltration revealed a significant increase in M2 macrophages within leiomyoma tissues compared to normal controls, highlighting a shift towards a pro-fibrotic immune profile.

Interestingly, our scRNA-seq trajectory analysis revealed a distinct, cell-type-specific divergent regulation of *CEBPD*. While *CEBPD* expression was strongly inversely correlated with the EMT and fibrotic progression score in fibroblasts, it exhibited a positive correlation within T lymphocytes. We hypothesize that in the stromal compartment, *CEBPD* acts as a critical molecular brake on collagen production, and its repression is fundamentally required for EMT progression and extracellular matrix hyper-secretion. Conversely, the concurrent upregulation of *CEBPD* in the immune compartment may reflect a reactive, compensatory response associated with localized T-cell activation or immune exhaustion within the increasingly rigid, hypoxic fibrotic tumor microenvironment. This duality underscores the complexity of *CEBPD*’s regulatory network across different cellular niches.

This finding aligns with the observed downregulation of *CEBPD*, suggesting its potential role in modulating macrophage polarization and activity (Tongjun et al., 2025). Our observation of CEBPD nuclear expression within CD3E-positive T lymphocytes in clinical ULM specimens raises important questions regarding its function in immune regulation, particularly the mechanisms by which it may shape T cell responses within the ULM tissue microenvironment. This immunomodulatory role of CEBPD warrants further in-depth investigation, as delineating the interactions between CEBPD and distinct immune cell populations may uncover novel therapeutic targets for the intervention and management of uterine leiomyoma (Nan et al., 2024).

The data obtained via multi-omics approaches allowed for a nuanced understanding of *CEBPD*’s mechanistic role in the context of fibrosis and epithelial-mesenchymal transition (EMT). Specifically, the dynamic regulation of *CEBPD* along the fibrosis axis indicates its potential as a negative regulator of fibrosis-associated gene expression (Meilian et al., 2021). The correlation between *CEBPD* expression and EMT scores in fibroblasts underscores the importance of cell-type specificity in determining *CEBPD*’s functional outcomes. Future research should focus on elucidating the downstream targets of *CEBPD* and the molecular pathways involved, particularly in fibroblasts and smooth muscle cells, which are critical in leiomyoma development (Kyle et al., 2022).

Given its central role as a negative regulator of fibrogenesis, *CEBPD* presents a compelling, pharmacologically actionable target for the non-surgical management of ULM. While transcription factors are classically considered challenging to drug directly, *CEBPD* expression can be modulated via upstream cytokine signaling networks or epigenetic interventions. For instance, specific cytokine therapies or repurposed epigenetic modulators, such as histone deacetylase (HDAC) inhibitors, have demonstrated efficacy in restoring *CEBPD* levels in other fibrotic and neoplastic contexts. Future pre-clinical studies should investigate whether targeted epigenetic reactivation of *CEBPD* can successfully arrest or reverse extracellular matrix deposition in uterine fibroids, thereby translating these transcriptomic findings into viable pharmacological strategies.

The present study, while providing valuable insights into the role of *CEBPD* in uterine leiomyoma, is not without limitations. Notably, the lack of functional wet-lab validation restricts the confirmation of *CEBPD*’s mechanistic role as a negative regulator of fibrosis. Additionally, the restricted sample sizes may hinder the generalizability of the findings to broader patient populations, as correlations between *CEBPD* expression and clinical parameters remain unexplored. These histological findings serve as a proof-of-concept spatial validation that requires future corroboration in large-cohort, multi-center studies. Furthermore, the potential impact of batch effects and dataset heterogeneity on the observed results warrants cautious interpretation, especially given the inconsistencies noted across different datasets. Lastly, while the single-cell RNA-seq analysis enriches our understanding of cellular heterogeneity within leiomyomas, it is imperative that these findings be validated in larger cohorts to substantiate the identified cell populations and expression dynamics.

## 5 Conclusion

In conclusion, this multi-omics investigation elucidates the downregulation of *CEBPD* as a critical feature of uterine leiomyoma, linking it to altered immune infiltration and fibrotic processes within the tumor microenvironment. The diagnostic potential of *CEBPD*, coupled with its proposed role in regulating epithelial-mesenchymal transition and collagen production, positions it as a promising biomarker and therapeutic target for further exploration. Future studies should aim to validate the functional implications of *CEBPD*, investigate its utility in diagnostics, and assess its therapeutic potential in disrupting the pro-fibrotic network characteristic of uterine leiomyomas.

## Data Availability

The original contributions presented in the study are included in the article/Supplementary Material, further inquiries can be directed to the corresponding authors.

## Supplementary material

SUPPLEMENTARY FIGURE S1 Quality control criteria for scRNA-seq data.

SUPPLEMENTARY FIGURE S2 R code for CellChat intercellular communication analysis.

SUPPLEMENTARY FIGURE S3 Enriched biological pathways in GSE64763 dataset.

SUPPLEMENTARY FIGURE S4 Enriched biological pathways in GSE95101 dataset.
