# Supplementary material for: CEBPD may function as a molecular indicator of fibrotic severity and negative regulator of fibrosis in uterine leiomyoma through regulating EMT progression
Source: Front Pharmacol. 2026 May 19;17:1839523. doi: 10.3389/fphar.2026.1839523 (PMC13226546; doi:10.3389/fphar.2026.1839523)
Supplement: Supplementary file 1 [file Supplementaryfile1.docx]

**
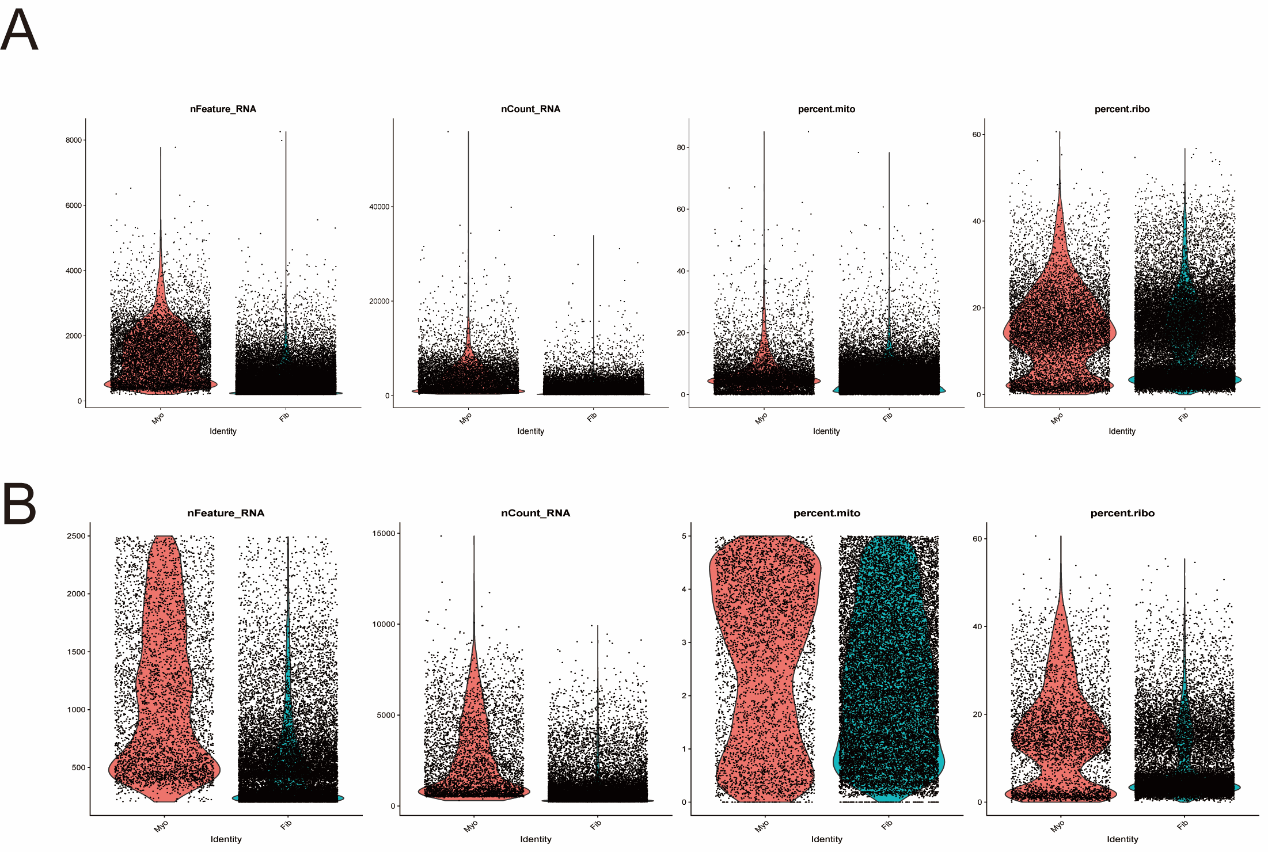
Supplementary Figure S1. Quality control metrics of single-cell RNA sequencing data.** (A) Violin plots displaying the distribution of key quality control parameters pre- and post-filtering across all integrated samples. Parameters include the number of unique features (nFeature_RNA), total molecule counts (nCount_RNA), and the percentage of mitochondrial transcripts (percent.mito). (B) A mitochondrial threshold of < 20% was applied to retain biologically viable stromal and smooth muscle cells that inherently exhibit elevated baseline stress signatures following robust tissue dissociation.
