# Supplementary material for: CEBPD may function as a molecular indicator of fibrotic severity and negative regulator of fibrosis in uterine leiomyoma through regulating EMT progression
Source: Front Pharmacol. 2026 May 19;17:1839523. doi: 10.3389/fphar.2026.1839523 (PMC13226546; doi:10.3389/fphar.2026.1839523)
Supplement: Supplementary file 2 [file Supplementaryfile2.docx]

options(stringsAsFactors = F)

library(Seurat)

library(ggplot2)

library(clustree)

library(cowplot)

library(dplyr)

library(data.table)

library(stringr)

library(DoubletFinder)

#install.packages("textshape")

library(textshape)

# 获取当前工作目录

current_dir <- getwd()

# 获取当前目录下所有子目录，这些子目录可作为样本

sample_dirs <- list.dirs(path = current_dir, full.names = TRUE, recursive = FALSE)

# 初始化一个空列表，用于存储每个样本的 Seurat 对象

seurat_objects <- list()

# 循环遍历每个样本目录

for (sample_dir in sample_dirs) {

# 提取样本名，即子目录名

sample_name <- basename(sample_dir)

# 尝试读取 10X 数据

tryCatch({

# 读取 10X 数据

sample_data <- Read10X(data.dir = sample_dir)

# 创建 Seurat 对象，添加了min.features和min.cells参数

seurat_obj <- CreateSeuratObject(counts = sample_data,

project = sample_name,

min.features = 200,

min.cells = 3)

# 将 Seurat 对象添加到列表中

seurat_objects[[sample_name]] <- seurat_obj

cat(paste0("成功读取样本 ", sample_name, " 的数据\n"))

}, error = function(e) {

cat(paste0("读取样本 ", sample_name, " 的数据时出错: ", conditionMessage(e), "\n"))

})

}

# 查看已读取的样本名

sample_names <- names(seurat_objects)

print(sample_names)

# 合并所有的 Seurat 对象

if (length(seurat_objects) > 0) {

# 以第一个 Seurat 对象为基础，将其余的依次合并

merged_seurat <- seurat_objects[[1]]

if (length(seurat_objects) > 1) {

for (i in 2:length(seurat_objects)) {

merged_seurat <- merge(x = merged_seurat, y = seurat_objects[[i]])

}

}

print("所有样本已成功合并。")

} else {

print("没有成功读取到任何样本数据，无法进行合并。")

}

####################祛除双细胞函数

detect_doublets <- function(seurat_obj) {

# 确保对象中包含orig.ident信息

if (!"orig.ident" %in% colnames(seurat_obj@meta.data)) {

stop("Metadata must contain 'orig.ident' column for multi-sample processing")

}

# 拆分对象为样本列表（使用orig.ident）

sample_list <- SplitObject(seurat_obj, split.by = "orig.ident")

# 创建空列表存储处理后的样本

processed_samples <- list()

# 遍历每个样本

for (sample_name in names(sample_list)) {

sample_obj <- sample_list[[sample_name]]

# 为当前样本创建临时副本 - 兼容Seurat v5的Assay5

# 方法1：直接使用GetAssayData获取counts数据

counts_data <- GetAssayData(sample_obj, assay = "RNA", layer = "counts")

# 方法2：使用layer参数创建新对象

temp_obj <- CreateSeuratObject(

counts = counts_data,

meta.data = sample_obj@meta.data

)

# 标准化和PCA用于双细胞检测

temp_obj <- NormalizeData(temp_obj)

temp_obj <- FindVariableFeatures(temp_obj, selection.method = "vst", nfeatures = 2000)

temp_obj <- ScaleData(temp_obj)

temp_obj <- RunPCA(temp_obj, npcs = 30, verbose = FALSE)

# 估计双细胞比例（10X数据通常为5-10%）

nExp <- round(ncol(temp_obj) * 0.075)

# 运行DoubletFinder

temp_obj <- doubletFinder(

temp_obj,

PCs = 1:10,

pN = 0.25,

pK = 0.09,

nExp = nExp,

reuse.pANN = NULL,

sct = FALSE

)

# 提取双细胞检测结果列名

df_col <- grep("DF.classifications", colnames(temp_obj@meta.data), value = TRUE)

# 统计并打印双细胞比例

n_doublets <- sum(temp_obj@meta.data[[df_col]] == "Doublet")

doublet_percent <- round(n_doublets / ncol(temp_obj) * 100, 2)

message("样本 ", sample_name, ": 检测到 ", n_doublets, " 个双细胞 (", doublet_percent, "%)")

# 在原始样本对象中添加双细胞信息

sample_obj@meta.data$doublet_status <- temp_obj@meta.data[[df_col]]

# 去除双细胞

sample_obj <- subset(sample_obj, subset = doublet_status == "Singlet")

# 移除临时添加的列

sample_obj@meta.data$doublet_status <- NULL

# 保存处理后的样本

processed_samples[[sample_name]] <- sample_obj

# 清理临时对象释放内存

rm(temp_obj)

gc()

}

# 合并所有样本

merged_obj <- merge(processed_samples[[1]], processed_samples[-1])

# 恢复原始元数据列

merged_obj@meta.data <- merged_obj@meta.data[, colnames(seurat_obj@meta.data)]

return(merged_obj)

}

sce<-merged_seurat

# 使用示例

sce_clean <- detect_doublets(sce)

sce<-sce_clean

# 假设 sce 是你的 Seurat 对象

sce$orig.ident <- ifelse(sce$orig.ident %in% c("GSM4942396","GSM4942397","GSM4942398","GSM4942399","GSM5023319"), "Myo",

ifelse(sce$orig.ident %in% c("GSM5023320","GSM5023321","GSM5023323","GSM5023324_1","GSM5023324_2","GSM6509142"), "Fib",sce$orig.ident))

unique(sce$orig.ident)

head(rownames(sce))

Idents(sce)<-sce$orig.ident

# 自动计算线粒体

sce[["percent.mito"]] <- PercentageFeatureSet(sce,pattern="^MT-")##注意MT为人类线粒体，Mt或mt为小鼠线粒体

# 自动计算核糖体

sce[["percent.ribo"]] <- PercentageFeatureSet(sce,pattern="^RP[SL]")#"^Rp[sl]"小鼠

#########线粒体、核糖体基因过滤前的数据绘制小提琴图，便于后续阈值选定##########

VlnPlot_G <- VlnPlot(sce, features = c("nFeature_RNA","nCount_RNA","percent.mito","percent.ribo"),ncol=4) + NoLegend()

VlnPlot_G #查看小提琴图

pdf("Vlnplot质控前.pdf", width =28, height = 8)

print(VlnPlot_G)

dev.off()

########绘制散点图，查看nCount_RNA、nFeature_RNA、percent.mito三者关系图#############

plot0_G <- FeatureScatter(sce, feature1 = "nFeature_RNA", feature2 = "percent.mito")

plot1_G <- FeatureScatter(sce, feature1 = "nCount_RNA", feature2 = "percent.mito")

plot2_G <- FeatureScatter(sce, feature1 = "nCount_RNA", feature2 = "nFeature_RNA")

plot0_G+plot1_G+plot2_G

pdf("散点图质控.pdf", width =16, height = 5)

print(plot0_G+plot1_G+plot2_G)

dev.off()

########根据线粒体，核糖体基因过滤不符合要求的细胞，percent.mito表示每个细胞中线粒体、核糖体基因##############

sce <- subset(sce, subset = nFeature_RNA > 200 & nFeature_RNA < 2500 & percent.mito < 5)

########绘制小提琴图查看过滤后情况####################

VlnPlot_G1 <- VlnPlot(sce, features = c("nFeature_RNA","nCount_RNA","percent.mito","percent.ribo"),ncol=4) + NoLegend()

VlnPlot_G1

pdf("Vlnplot质控后.pdf", width =24, height = 8)

print(VlnPlot_G1)

dev.off()

sce1<-sce

sce<-JoinLayers(sce)

#sce <- SeuratObject::UpdateSeuratObject(sce)

# 提取原始UMI计数矩阵（假设存储在'assay'中，默认为RNA）

umi_counts <- GetAssayData(sce, layer = "counts")

# 计算每个细胞的UMI总数（对矩阵的每一列求和）

umi_per_cell <- colSums(umi_counts)

# 计算UMI计数的中位数

umi_median <- median(umi_per_cell)

# 输出结果

cat("UMI计数中位数:", umi_median, "\n")

# 判断中位数是否低于1,000或高于50,000

if (umi_median < 1000) {

cat("警告：UMI计数中位数低于1,000，考虑降低scale.factor\n")

} else if (umi_median > 50000) {

cat("警告：UMI计数中位数高于50,000，考虑增加scale.factor\n")

} else {

cat("UMI计数中位数在正常范围内，可使用默认scale.factor (1e4)\n")

}

sce <- NormalizeData(sce,

normalization.method = "LogNormalize",

scale.factor = 1e4)

sce <- FindVariableFeatures(sce)

top10_G <- head(VariableFeatures(sce), 10)

plot_hight_G_1 <- VariableFeaturePlot(sce)

plot_hight_G_2 <- LabelPoints(plot =plot_hight_G_1, points = top10_G, repel = TRUE,xnudge=0,ynudge=0)

pdf("高变基因.pdf", width =7, height = 5)

print(plot_hight_G_2)

dev.off()

sce <- ScaleData(sce)

sce <- RunPCA(sce, features = VariableFeatures(object = sce))

##对降维结果绘图

pdf("VizDimLoadings确定分群.pdf", width =50, height = 50)

VizDimLoadings(sce, dims = 1:40, reduction = "pca")

dev.off()

p1<-DimPlot(sce,reduction = "pca")##对降维结果绘图

pdf("DimPlot_PCA.pdf", width =7, height = 5)

print(p1)

dev.off()

##对降维结果绘制热图

pdf("DimHeatmap确定分群.pdf", width =50, height = 50)

DimHeatmap(sce, dims = 1:40, cells = 500, balanced = TRUE)

dev.off()

#install.packages("harmony")

library(harmony)

seuratObj <- RunHarmony(sce, "orig.ident")

names(seuratObj@reductions)

pdf("ElbowPlot确定分群.pdf", width =15, height = 8)

ElbowPlot(seuratObj,reduction = 'harmony',ndims = 50)

dev.off()

seuratObj <- FindNeighbors(seuratObj, reduction = "harmony",

dims = 1:10)

seuratObj1<-seuratObj

for (res in c(0.1,0.2,0.3,0.4,0.5,0.6,0.7,0.8,0.9,1.0,1.1,1.2,1.3,1.4,1.5,1.6)) {

seuratObj1=FindClusters(seuratObj1, graph.name = "RNA_snn", resolution = res, algorithm = 1)}

apply(seuratObj1@meta.data[,grep("RNA_snn_res",colnames(seuratObj1@meta.data))],2,table)

p2_tree=clustree(seuratObj1@meta.data, prefix = "RNA_snn_res.")

pdf(file = "10.clustertree.pdf",width =14,height =16)

p2_tree

dev.off()

seuratObj1=FindClusters(seuratObj, #graph.name = "CCA_snn",

resolution = 1, algorithm = 1)

seuratObj1 <- RunUMAP(seuratObj1, dims = 1:10,

reduction = "harmony")

seuratObj1 <- RunTSNE(seuratObj1, dims = 1:10, reduction = "harmony")

p5<-DimPlot(seuratObj1,reduction = "umap",label=T)

pdf("DimPlot_clusters_umap.pdf", width =7.5, height = 5.5)

print(p5)

dev.off()

p5<-DimPlot(seuratObj1,reduction = "umap",label=T,split.by = "orig.ident")

pdf("DimPlot_clusters_umap_bygroup.pdf", width =12.5, height = 5.5)

print(p5)

dev.off()

seuratObj1<-JoinLayers(seuratObj1)

saveRDS(seuratObj1,"clusters.rds")

combined.markers <- FindAllMarkers(seuratObj1,only.pos = TRUE, min.pct = 0.7, logfc.threshold = 0.6)

top100 <- combined.markers %>% group_by(cluster) %>% top_n(n = 100, wt = avg_log2FC)

write.csv(top100,"diffexpr_to100.csv")

write.csv(combined.markers,"diffexpr_clusters.csv")

seuratObj1$celltype<- plyr::mapvalues(seuratObj1$seurat_clusters,

from = 0:25,

to=c(

"SMC",

"Fib",

"SMC",

"NK",

"T",

"NK",

"Fib",

"Fib",

"End",

"SMC",

"Mac",

"Fib",

"Mac",

"Fib",

"End",

"End",

"SMC",

"T",

"Other",

"T",

"Other",

"Epi",

"SMC",

"SMC",

"Other",

"Other"

))

Idents(seuratObj1)<-seuratObj1$celltype

saveRDS(seuratObj1,"细胞注释之后.rds")

c <- c("#a2d2e7", "#f36569", "#67a8cd", "#ffc17f", "#cf9f88", "#6fb3a8",

"#b3e19b", "#50aa4b", "#ff9d9f",

"#cdb6da", "#704ba1", "#9a7fbd", "#dba9a4", "#e43030",

"#e99b78", "#ff8831","#6803c9","#41214c","#0000FF","#d6e2d2","#7CFC00","#979598","#F01780","#1E90fd","#ddFdfF","#FFFFE0","#fdcde6","#FF5500")

p6<-scCustomize::DimPlot_scCustom(seuratObj1,reduction = "umap", figure_plot = TRUE,label = F,colors_use = c)

pdf("DimPlot_celltype_umap.pdf", width =7, height = 5)

print(p6)

dev.off()

p6<-scCustomize::DimPlot_scCustom(seuratObj1, reduction = "umap", figure_plot = TRUE,label = F,colors_use = c,split.by = "orig.ident")

pdf("DimPlot_celltype_umap_bygroup.pdf", width =12, height = 5)

print(p6)

dev.off()

p7<- FeaturePlot(seuratObj1, features = "CEBPD")

pdf("CEBPD_umap_bygroup.pdf", width =12, height = 10)

print(p7)

dev.off()

combined.markers <- FindAllMarkers(seuratObj1,only.pos = TRUE, min.pct = 0.5, logfc.threshold = 0.36)

top4 <- combined.markers %>% group_by(cluster) %>% top_n(n = 4, wt = avg_log2FC)

p7<-DotPlot(seuratObj1, features = unique(top4$gene)) + RotatedAxis()

pdf("Dotplot_marker.pdf", width =18, height = 8)

print(p7)

dev.off()

write.csv(combined.markers,"combined.markers.csv")

pdf("Heatmap_marker.pdf", width =18, height = 18)

DoHeatmap(seuratObj1, features = unique(top4$gene), size = 4.7, angle = 45) +

NoLegend() +

scale_fill_gradient(low = "#fdfde1", high = "#064d59") +

theme(plot.margin = unit(c(6, 6, 6, 6), "cm"))

dev.off()

#########################柱状图

sample_table <- as.data.frame(table(seuratObj1@meta.data$orig.ident,seuratObj1@meta.data$celltype))

names(sample_table ) <- c("Samples","celltype","CellNumber")

# 计算每个样本中每种细胞类型的百分比

sample_table <- sample_table %>%

group_by(Samples) %>%

mutate(CellPercentage = CellNumber / sum(CellNumber))

# 规定样本顺序

sample_table$Samples<-factor(sample_table$Samples,levels = c("Myo","Fib"))

pp <- ggplot(sample_table, aes(x = Samples, y = CellPercentage, fill = celltype,

stratum = celltype, alluvium = celltype)) +

scale_fill_manual(values = c) +

scale_y_continuous(labels = scales::percent, expand = c(0, 0)) + # 将y轴显示为百分比

theme_classic()

library(ggalluvial)

# 添加柱状图和流动连接线

p2 <- pp +

geom_col(width = 0.6, color = NA) +

geom_flow(width = 0.6, alpha = 0.22, knot.pos = 0)

p2

pdf("cellpercentage.pdf", width =7, height = 8)

print(p2)

dev.off()

################################################################################

# 深入分析 CEBPD 在 Myo 与 Fib 组间的差异

################################################################################

# 确保加载了必要的库

library(Seurat)

library(ggplot2)

library(dplyr)

# 1. CEBPD 在两组间的空间分布对比 (Split FeaturePlot)

# -------------------------------------------------------------------------

p_split <- FeaturePlot(seuratObj1, features = "CEBPD", split.by = "orig.ident",

cols = c("lightgrey", "#DE2D26"), order = TRUE, pt.size = 0.5) &

theme(legend.position = "right")

pdf("CEBPD_Spatial_Comparison.pdf", width = 12, height = 5)

print(p_split)

dev.off()

# 2. 细胞类型特异性的 CEBPD 表达对比 (VlnPlot + Boxplot)

# -------------------------------------------------------------------------

# 使用 VlnPlot 比较不同细胞类型中 CEBPD 在两组间的表达量

p_vln <- VlnPlot(seuratObj1, features = "CEBPD", split.by = "orig.ident",

group.by = "celltype", pt.size = 0, combine = TRUE) +

geom_boxplot(width = 0.15, fill = "white", outlier.shape = NA, position = position_dodge(0.9)) +

theme_light() +

labs(title = "CEBPD Expression across Cell Types: Myo vs Fib")

pdf("CEBPD_CellType_VlnPlot.pdf", width = 14, height = 7)

print(p_vln)

dev.off()

# 3. 计算 CEBPD 阳性细胞的比例 (Percentage Analysis)

# -------------------------------------------------------------------------

# 统计在每个样本/组中，CEBPD 表达量 > 0 的细胞占比

exp_data <- FetchData(seuratObj1, vars = c("CEBPD", "orig.ident", "celltype"))

percent_stats <- exp_data %>%

group_by(orig.ident, celltype) %>%

summarize(

Total_Cells = n(),

CEBPD_Pos_Cells = sum(CEBPD > 0),

Percent_Positive = (CEBPD_Pos_Cells / Total_Cells) * 100

)

write.csv(percent_stats, "CEBPD_Positive_Percentage_Stats.csv")

# 4. CEBPD 与其潜在靶基因的相关性分析 (Correlation)

# -------------------------------------------------------------------------

# CEBPD 是转录因子，通常与炎症或应激基因共表达

# 提取表达矩阵

expr_matrix <- GetAssayData(seuratObj1, layer = "data")

cebpd_vector <- as.numeric(expr_matrix["CEBPD", ])

# 计算与前 2000 个高变基因的相关性

hvg <- VariableFeatures(seuratObj1)

cor_values <- apply(expr_matrix[hvg, ], 1, function(x) cor(as.numeric(x), cebpd_vector))

top_cor_genes <- sort(cor_values, decreasing = TRUE)[2:11] # 排除 CEBPD 自身

cat("与 CEBPD 相关性最强的基因有: \n")

print(names(top_cor_genes))

# 5. 组间差异显著性测试 (修正版)

# -------------------------------------------------------------------------

# 目标：在 "Fib" 细胞群中，对比 Myo 组和 Fib 组的 CEBPD 表达差异

if ("Fib" %in% seuratObj1$celltype) {

# 提取成纤维细胞子集

fib_subset <- subset(seuratObj1, idents = "Fib")

# 核心修正：指定 group.by = "orig.ident"，告诉函数去这个列里找 Myo 和 Fib

de_stats <- FindMarkers(fib_subset,

ident.1 = "Myo",

ident.2 = "Fib",

group.by = "orig.ident", # 必须加上这一行

features = "CEBPD",

logfc.threshold = 0,

test.use = "wilcox") # 使用默认的威尔科克森秩和检验

print(de_stats)

write.csv(de_stats, "CEBPD_DE_Test_in_Fib_Subset.csv")

}

# 批量计算所有细胞类型中 CEBPD 的组间差异

all_celltypes <- levels(seuratObj1$celltype)

diff_summary <- data.frame()

for (ct in all_celltypes) {

sub_obj <- subset(seuratObj1, idents = ct)

# 检查该细胞类型是否在两组中都存在，且细胞数大于 3

if (length(unique(sub_obj$orig.ident)) == 2 && min(table(sub_obj$orig.ident)) > 3) {

res <- FindMarkers(sub_obj, ident.1 = "Myo", ident.2 = "Fib",

group.by = "orig.ident", features = "CEBPD",

logfc.threshold = 0)

res$celltype <- ct

res$gene <- rownames(res)

diff_summary <- rbind(diff_summary, res)

}

}

# 按照 Log2FoldChange 排序，看哪种细胞中 CEBPD 变化最剧烈

diff_summary <- diff_summary %>% arrange(desc(abs(avg_log2FC)))

write.csv(diff_summary, "CEBPD_LogFC_Across_All_CellTypes.csv")

# 可视化这些差异

library(ggpubr)

p_final <- ggplot(diff_summary, aes(x = reorder(celltype, avg_log2FC), y = avg_log2FC, fill = avg_log2FC > 0)) +

geom_bar(stat = "identity") +

coord_flip() +

scale_fill_manual(values = c("skyblue", "tomato"), labels = c("Fib High", "Myo High")) +

labs(title = "CEBPD Differential Expression (Myo vs Fib)", x = "Cell Type", y = "Log2 Fold Change") +

theme_minimal()

pdf("CEBPD_LogFC_Barplot.pdf", width = 8, height = 6)

print(p_final)

dev.off()

################################################################################

# 5. CellChat 分析修正

################################################################################

library(CellChat)

library(patchwork)

library(ggalluvial)

library(Seurat)

# 确保 orig.ident 包含 Myo 和 Fib 组别信息

# 如果你想对比两组，通常需要分别构建对象再合并，或者在 meta 中标记

data.input <- GetAssayData(seuratObj1, assay = "RNA", layer = "data")

# 这里的 group.by 设为 celltype

cellchat <- createCellChat(object = data.input, meta = seuratObj1@meta.data, group.by = "celltype")

# 设置参考数据库

CellChatDB <- CellChatDB.human

cellchat@DB <- CellChatDB

# 预处理：抽样（如果细胞数过万，建议 subsetData 后抽样以加快速度）

cellchat <- subsetData(cellchat)

cellchat <- identifyOverExpressedGenes(cellchat)

cellchat <- identifyOverExpressedInteractions(cellchat)

# 计算通信概率 (如果报错，尝试设置 nboot = 20 减少计算量)

cellchat <- computeCommunProb(cellchat, raw.use = TRUE)

cellchat <- filterCommunication(cellchat, min.cells = 10)

cellchat <- computeCommunProbPathway(cellchat)

cellchat <- aggregateNet(cellchat)

# 可视化部分

pathways_all <- cellchat@netP$pathways

target_pathways <- intersect(c("COLLAGEN", "FN1", "THBS", "SPP1", "CCL"), pathways_all)

if (length(target_pathways) > 0) {

for (pathway in target_pathways) {

pdf(paste0("CellChat_", pathway, "_Network.pdf"), width = 8, height = 8)

netVisual_aggregate(cellchat, signaling = pathway, layout = "circle")

dev.off()

}

}

# 计算中心性

cellchat <- netAnalysis_computeCentrality(cellchat, slot.name = "netP")

################################################################################

################################################################################

# 1. 环境准备与基因集加载 (离线版)

################################################################################

library(GSVA)

library(GSEABase)

library(limma)

library(Seurat)

library(patchwork)

# 自动检测本地 GMT 文件

gmt_files_present <- list.files(pattern = "\\.gmt$")

if (length(gmt_files_present) > 0) {

actual_gmt <- gmt_files_present[1]

message("检测到文件: ", actual_gmt)

geneset <- getGmt(actual_gmt)

fgsea_sets <- geneIds(geneset)

} else {

stop("未发现GMT文件，请确保 h.all.v2023.2.Hs.symbols.gmt 在工作目录下")

}

################################################################################

# 2. GSVA 运行 (适配新版 API 2.0+)

################################################################################

# 内存优化：抽样

Idents(seuratObj1) <- "celltype"

sce_gsva_sub <- subset(seuratObj1, downsample = 200)

# 准备矩阵

expr_gsva <- as.matrix(GetAssayData(sce_gsva_sub, layer = "data"))

# --- 核心修正：使用新版 Param 对象 ---

# 使用 ssgseaParam 替代旧的 gsva() 参数写法

ssgsea_params <- ssgseaParam(expr_gsva, fgsea_sets)

gsva_res <- gsva(ssgsea_params)

# 将结果转置并存入 metadata

gsva_df <- as.data.frame(t(gsva_res))

# 关键步骤：统一处理列名，防止后续 limma 报错

colnames(gsva_df) <- gsub("-", "_", colnames(gsva_df))

sce_gsva_sub <- AddMetaData(sce_gsva_sub, metadata = gsva_df)

cat("GSVA 计算完成。\n")

################################################################################

# 3. Limma 差异通路分析 (修正列名匹配报错)

################################################################################

# 以 Fib 细胞为例

sce_fib <- subset(sce_gsva_sub, idents = "Fib")

group <- factor(sce_fib$orig.ident) # 需包含 Myo 和 Fib 两组

design <- model.matrix(~0 + group)

colnames(design) <- levels(group)

# --- 核心修正：动态匹配 metadata 中的通路列名 ---

# 此时 meta.data 中的列名已经过 gsub 处理，需与处理后的名称对应

actual_pathway_names <- gsub("-", "_", names(fgsea_sets))

# 只提取确实存在于 metadata 中的 Hallmark 列

valid_columns <- intersect(actual_pathway_names, colnames(sce_fib@meta.data))

gsva_mat <- t(as.matrix(sce_fib@meta.data[, valid_columns]))

# 差异运算

fit <- lmFit(gsva_mat, design)

# 注意：确保你的 levels 顺序，这里设定对比为 Myo 比 Fib

contrast.matrix <- makeContrasts(Myo - Fib, levels = design)

fit2 <- contrasts.fit(fit, contrast.matrix)

fit2 <- eBayes(fit2)

pathway_diff <- topTable(fit2, coef = 1, number = Inf)

write.csv(pathway_diff, "GSVA_Limma_Pathways_Myo_vs_Fib_in_FibCells.csv")

################################################################################

# 4. 可视化与轨迹分析

################################################################################

# 通路评分分布图

target_pathways <- intersect(colnames(sce_gsva_sub@meta.data),

c("HALLMARK_INFLAMMATORY_RESPONSE",

"HALLMARK_HYPOXIA",

"HALLMARK_TGF_BETA_SIGNALING"))

if(length(target_pathways) > 0){

pdf("GSVA_Pathway_VlnPlot.pdf", width = 12, height = 8)

print(VlnPlot(sce_gsva_sub, features = target_pathways, split.by = "orig.ident",

group.by = "celltype", pt.size = 0))

dev.off()

}

# 1. 提取绘图数据

# 假设我们用已算出的 HALLMARK_EPITHELIAL_MESENCHYMAL_TRANSITION (EMT) 作为轴

pseudo_axis <- "HALLMARK_EPITHELIAL_MESENCHYMAL_TRANSITION"

# 检查该通路是否存在于 meta.data

if(!pseudo_axis %in% colnames(sce_gsva_sub@meta.data)){

# 如果名字被处理过，尝试自动匹配

pseudo_axis <- grep("EPITHELIAL_MESENCHYMAL", colnames(sce_gsva_sub@meta.data), value = TRUE)[1]

}

plot_df <- data.frame(

Pseudotime_Score = sce_gsva_sub@meta.data[[pseudo_axis]],

CEBPD_Exp = GetAssayData(sce_gsva_sub, layer = "data")["CEBPD", ],

CellType = sce_gsva_sub$celltype,

Group = sce_gsva_sub$orig.ident

)

# 2. 绘制趋势图

library(ggplot2)

library(ggpubr)

p_trend <- ggplot(plot_df, aes(x = Pseudotime_Score, y = CEBPD_Exp)) +

# 背景散点

geom_point(aes(color = Group), alpha = 0.2, size = 0.5) +

# 拟合曲线（展示随通路评分增加，CEBPD 的动态变化）

geom_smooth(method = "gam", color = "black", se = TRUE) +

scale_color_manual(values = c("Myo" = "#2166ac", "Fib" = "#b2182b")) +

facet_wrap(~CellType, scales = "free_y") +

theme_minimal() +

labs(x = paste("Progression Axis:", pseudo_axis),

y = "CEBPD Expression Level",

title = "CEBPD Dynamics along Fibrosis Progression") +

stat_cor(method = "pearson", label.x.npc = "middle") # 添加相关系数

pdf("CEBPD_Progression_Trend.pdf", width = 12, height = 8)

print(p_trend)

dev.off()

library(ComplexHeatmap)

library(circlize)

# 1. 准备数据：提取成纤维细胞并排序

sce_fib <- subset(sce_gsva_sub, idents = "Fib")

# 使用 EMT 通路评分作为排序依据（即我们的“伪拟时序”）

pseudo_order <- order(sce_fib$HALLMARK_EPITHELIAL_MESENCHYMAL_TRANSITION)

sce_fib_sorted <- sce_fib[, pseudo_order]

# 2. 选取要展示的基因

# 包含你筛选出的保护性基因和典型的纤维化基因

genes_to_show <- c("CEBPD", "KLF2", "NR4A1", "ZFP36", # 保护/稳态基因 (Myo高表达)

"COL1A1", "COL1A2", "COL3A1", "ACTA2", "MALAT1") # 纤维化基因 (Fib高表达)

# 3. 提取表达矩阵并进行平滑处理 (Rolling Mean)

# 平滑处理能让热图看起来像 Monocle 一样丝滑，减少单细胞的噪音

exp_mat <- as.matrix(GetAssayData(sce_fib_sorted, layer = "data")[genes_to_show, ])

# 定义一个简单的滑动窗口平滑函数

smooth_mat <- t(apply(exp_mat, 1, function(x) {

zoo::rollmean(x, k = 50, fill = "extend") # k值越大越丝滑

}))

# 4. 归一化 (Z-score) 方便对比趋势

smooth_mat_scaled <- t(scale(t(smooth_mat)))

# 5. 绘制热图

col_fun = colorRamp2(c(-2, 0, 2), c("#2166ac", "white", "#b2182b"))

pdf("Fibrosis_Progression_Heatmap_NoMonocle.pdf", width = 10, height = 6)

Heatmap(smooth_mat_scaled,

name = "Z-score Expression",

cluster_columns = FALSE, # 保持按评分排序

cluster_rows = TRUE,

show_column_names = FALSE,

col = col_fun,

column_title = "Fibrosis Progression (Low EMT Score -> High EMT Score)",

row_names_side = "left",

# 添加顶部注释：展示组别分布

top_annotation = HeatmapAnnotation(

Group = sce_fib_sorted$orig.ident,

col = list(Group = c("Myo" = "#66c2a5", "Fib" = "#fc8d62"))

))

dev.off()

# SCI 标准配色方案（推荐 Nature 风格）

sci_colors <- c("#E64B35FF", "#4DBBD5FF", "#00A087FF", "#3C8DBCFF", "#F39C12FF", "#8E44ADFF", "#7F8C8DFF", "#2ECC71FF")

# 定义全局主题

sci_theme <- theme_bw() +

theme(

panel.grid = element_blank(),

text = element_text(family = "Arial", size = 10),

axis.title = element_text(size = 12, face = "bold"),

axis.text = element_text(size = 10, color = "black"),

legend.title = element_text(size = 10, face = "bold"),

plot.title = element_text(size = 13, face = "bold", hjust = 0.5),

strip.background = element_rect(fill = "white", color = "white"),

strip.text = element_text(size = 12, face = "bold")

)

# 优化 UMAP 绘图

p6 <- scCustomize::DimPlot_scCustom(seuratObj1, reduction = "umap",

figure_plot = TRUE, label = FALSE,

colors_use = c, pt.size = 0.6) +

sci_theme +

labs(title = "Single-cell Landscape of Uterus") # 增加更有学术感的标题

# 保存为 PDF (矢量图) 和 TIFF (300DPI)

ggsave("Figure1_UMAP_Celltype.pdf", p6, width = 7, height = 6)

# 如果需要位图，使用以下命令：

# ggsave("Figure1_UMAP_Celltype.tiff", p6, width = 7, height = 6, dpi = 300, compression = "lzw")

# 兼容性 SCI 主题：移除特定的 Arial 调用，改用系统默认

sci_theme_fixed <- theme_bw() +

theme(

panel.grid.major = element_blank(),

panel.grid.minor = element_blank(),

legend.position = "right",

text = element_text(family = ""), # 留空，使用系统默认字体，防止报错

axis.text = element_text(size = 10, color = "black"),

axis.title = element_text(size = 12, face = "bold"),

strip.background = element_rect(fill = "grey95", color = NA),

strip.text = element_text(size = 11, face = "bold"),

plot.title = element_text(size = 14, face = "bold", hjust = 0.5)

)

library(ggplot2)

library(ggpubr)

p_trend_opt <- ggplot(plot_df, aes(x = Pseudotime_Score, y = CEBPD_Exp)) +

# 1. 降低背景点密度，突出趋势

geom_point(aes(color = Group), alpha = 0.1, size = 0.4) +

# 2. 增强趋势线，明确指定 GAM 公式以消除警告

geom_smooth(method = "gam", formula = y ~ s(x, bs = "cs"),

color = "black", fill = "#D9D9D9", se = TRUE, size = 1) +

# 3. SCI 经典配色（Myo 蓝 vs Fib 红）

scale_color_manual(values = c("Myo" = "#2166ac", "Fib" = "#b2182b")) +

# 4. 分面美化

facet_wrap(~CellType, scales = "free_y", ncol = 4) +

sci_theme_fixed +

# 5. 学术化坐标轴标签

labs(x = "EMT Progression Score (Pseudotime Proxy)",

y = "Log-normalized Expression (CEBPD)",

title = "Dynamic Expression of CEBPD along Fibrosis Axis") +

# 6. 相关系数精修

stat_cor(method = "pearson", size = 3.5, label.x.npc = "left", label.y.npc = "top")

# 7. 保存 PDF（使用 standard 字体映射，避开 Windows 字体冲突）

pdf("Figure5_CEBPD_Trend_Fixed.pdf", width = 12, height = 7, useDingbats = FALSE)

print(p_trend_opt)

dev.off()
